# “It is like a mind attack”*:* stress and coping among urban school-going adolescents in India

**DOI:** 10.1186/s40359-019-0306-z

**Published:** 2019-05-28

**Authors:** Rachana Parikh, Mahima Sapru, Madhuri Krishna, Pim Cuijpers, Vikram Patel, Daniel Michelson

**Affiliations:** 1 Sangath, C-1/52, 1st Floor, Safdarjung Development Area, New Delhi, Delhi, 110016 India; 2 Department of Clinical, Neuro and Developmental Psychology, Amsterdam Public Health research institute, Vrije Universiteit Amsterdam, van der Boechorstraat 1, 1081 BT Amsterdam, The Netherlands; 3 Present Address: Evalueserve.com Private Limited, Tower 6, 8th Floor, Candor Gurgaon One Realty Projects Pvt. Ltd., IT/ITES SEZ, Candor TechSpace, Tikri, Sector-48, Gurgaon, 122001 Haryana India; 4 Department of Global Health and Social Medicine, The Harvard TH Chan School of Public Health, Harvard Medical School, 641, Huntington Avenue, Boston, MA 02115 USA; 5 School of Psychology, University of Sussex, Falmer, Brighton, BN1 9RH UK

**Keywords:** Schools, Mental health, Stress, Coping, India

## Abstract

**Background:**

Mental health problems are leading contributors to the global disease burden in adolescents. This study aims to highlight (1) salient context-specific factors that influence stress and coping among school-going adolescents across two urban sites in India; and (2) potential targets for preventing mental health difficulties.

**Methods:**

Focus group discussions were undertaken with a large sample of 191 school-going adolescent boys and girls aged 11–17 years (mean = 14 years), recruited from low- and middle-income communities in the predominantly urban states of Goa and Delhi. Framework analysis was used to identify themes related to causes of stress, stress reactions, impacts and coping strategies.

**Results:**

Proximal social environments (home, school, peers and neighborhood) played a major role in causing stress in adolescents’ daily lives. Salient social stressors included academic pressure, difficulties in romantic relationships, negotiating parental and peer influences, and exposure to violence and other threats to personal safety. Additionally, girls highlighted stress from having to conform to normative gender roles and in managing the risk of sexual harassment, especially in Delhi. Anger, rumination and loss of concentration were commonly experienced stress reactions. Adolescents primarily used emotion-focused coping strategies (e.g., distraction, escape-avoidance, emotional support seeking). Problem-focused coping (e.g., instrumental support seeking) was less common. Examples of harmful coping (e.g., substance use) were also reported.

**Conclusions:**

The development of culturally sensitive and age-appropriate psychosocial interventions for distressed adolescents should attend to the challenges posed by home, school, peer and neighborhood environments. Enhancements to problem- and emotion-focused strategies are needed in order to bolster adolescents’ repertoire of adaptive coping skills in stressful social environments.

**Electronic supplementary material:**

The online version of this article (10.1186/s40359-019-0306-z) contains supplementary material, which is available to authorized users.

## Background

Adolescence is often described as a period of “storm and stress” [1], marked by increased susceptibility to mental disorders. Early identification and successful management of mental health problems in the adolescent years can improve long-term health outcomes and social adjustment [2]. Such efforts require an in-depth understanding of environmental risks, signs and idioms of psychological distress, and coping strategies for vulnerable youth across different contexts.

The psychological outcomes of an individual’s interactions with his or her environment can be understood through Lazarus and Folkman’s “stress-coping” theory [3]. In particular, an imbalance between internal/external demands and the perceived resources to deal with these challenges leads to negative emotional responses. Specific outcomes are mediated by appraisals of events in terms of perceived threat, control and access to coping resources. A persistent imbalance in this transactional stress-coping system contributes to the development and maintenance of a range of mental disorders, including both internalizing and externalizing difficulties [4, 5].

The majority of the world’s adolescents live in low- and middle-income countries (LMICs), where they are exposed to a range of psychosocial adversities [6]. India alone is home to more than 250 million adolescents aged 10–19 years, or 20% of the global adolescent population [7]. The National Mental Health Survey (2016) estimated that 13.3% of all adolescents residing in metropolitan areas have “mental morbidity,” double the prevalence in rural areas [8]. Correspondingly, studies conducted among school-going adolescents in urban India indicate that at least one in five adolescents endure high stress levels in their daily lives [9–13]. Although the relative importance of stressors differs across studies, commonly identified examples include academic pressure, adverse family events, educational/career concerns, challenges in romantic and sexual encounters, and navigating peer group dynamics [9, 14–16]. Adolescents reportedly adopt a wide range of coping strategies including problem solving, seeking support from parents and friends, praying, positive reframing, distraction, and avoidance [9, 14, 17].

Much of this surveyed literature from India is based on small and non-representative samples. The available studies provide little by way of in-depth exploration of key environmental stressors, impacts and mitigating strategies across different ages, genders and localities. A nuanced understanding of such contextual factors is essential for identifying intervention components that are culturally relevant and acceptable. In addition, in-depth knowledge of the local ecological context is needed for cultural adaptation of treatments proven to be effective elsewhere (e.g. through the inclusion of local metaphors). This is especially important in low- and middle-income countries such as India, where there is a relatively scarce local evidence base on adolescent mental health interventions.

The current study attempted to address this knowledge gap by using qualitative methods to explore: 1) common ecological stressors faced by adolescents in two predominantly urban states in India; 2) adolescents’ subjective experiences of stress; and 3) strategies used by adolescents to manage stress reactions across age, gender and sites. The ultimate aim was to provide contextually relevant insights for developing mental health interventions in Indian schools. A pragmatic approach was adopted to match the methods to study objectives, guided by principles of interpretivism and reflexivity [18, 19]. We used semi-structured focus group discussions with a large sample, allowing for variation in age, gender and geographic location. This permitted sensitive inquiry across diverse perspectives. For analysis, we employed a structured framework approach for thematic analysis, which has been widely used in other applied health and psychology research [20, 21]. The study is part of a larger research program (PRIDE), which seeks to develop and evaluate a suite of psychological interventions for common mental health problems in school-going adolescents in India [22].

## Methods

### Design and setting

This exploratory qualitative study was conducted in Delhi (India’s capital) and Goa, the country’s most highly urbanized state [7]. The methods have been reported in line with the consolidated criteria for reporting qualitative studies - COREQ [23]. A completed COREQ checklist for this study has been provided among the supplementary materials (Additional file 1 - COREQ checklist).

Participating students in Delhi were drawn from eight Hindi-medium high schools, run by the Delhi Government, and one English-medium private sector school. The Government schools were relatively large (with an average population of 2800 students across grades 6–12), providing single-gender education in low-income areas. The private-sector school provided co-education in a middle-class locality. In Goa, participating students were drawn from seven high schools (classes 5–10), run by the Archdiocese Board of Education. These schools were relatively small (with an average population of 500 students) and provided co-education in Konkani and English in middle-class localities.

### Sample

We conducted 22 focus group discussions (FGDs; Delhi = 12 and Goa = 10) with *N* = 191 adolescents (*n* = 112 girls, *n* = 79 boys; *n* = 108 in Delhi, *n* = 83 in Goa). Each focus group included 5–16 participants (median = 9), purposively sampled to maximize variation across age, gender and sites (Table 1). Participants ranged in age from 11 to 17 years, with students of similar age grouped together. Separate boys, girls and mixed groups were organized and participants within a given group often knew each other. Adolescents were invited to participate through classroom announcements by researchers and visits by researchers to community-based youth organizations working with adolescents from the participating schools. Representativeness was addressed by continuously monitoring participation rates across age, gender and site. Rates of non-participation were not systematically assessed, since recruitment activities focused on classrooms rather than individuals.

**Table 1:** Sample characteristics of the participants of the study

Sub-sample (organised by age group)	No. of FGDs	Boys (n)	Girls (n)	Total (N)
Delhi (11–14 years)	7	18	53	71
Delhi (15–17 years)	5	17	20	37
Goa (11–14 years)	7	26	25	51
Goa (15–17 years)	3	18	14	32
Total	22	79 (41%)	112 (59%)	191 (100%)

Adolescents who expressed an interest in participating were provided with a printed information sheet containing details about study aims and methods. A parallel parent version of the information sheet was distributed when adolescents were aged under 18 years. Prior written informed consent was obtained from all adolescents, and additional passive parental consent (active opting out of research) was obtained for all participating adolescents. The consent process and other study procedures were conducted in accordance with protocols approved by Institutional Review Boards at the Public Health Foundation of India (Ref:TRC-IEC-275/15), Sangath (Ref:VP_2015_017), Indian Council of Medical Research (Ref:HMSC/1/2016-SBR) and London School of Hygiene and Tropical Medicine (Ref:11967). Additional approvals were obtained from the Directorate of Education (Delhi) and Archdiocese Board of Education (Goa).

### Data collection

A semi-structured interview guide was developed specifically for this study, including open-ended questions on causes/experiences of stress and use of coping strategies (see supplementary materials, Additional File 2). Additional questions explored preferences for counselling and self-help interventions, findings for which are reported elsewhere [24]. Two researchers (usually RP and MS; both females and holding postgraduate degrees in public health) co-facilitated each FGD over 45–60 min. One researcher moderated the discussion, while the second researcher maintained notes and asked clarifying questions. Other interviewers (see Acknowledgments) included both males and females. FGDs were conducted in Hindi (12), English (9) and Konkani (1). All but two FGDs were audio-recorded, as administrators at the private-sector school denied permission for audio-recording. All audio-recordings were transcribed verbatim. The sole Konkani FGD was further translated into English, as none of the coders were Konkani speakers. We analyzed detailed notes from the two FGDs which were not audio-recorded. Data saturation was discussed within the team on an ongoing basis. Interim FGD summaries were continuously monitored for emergent themes by the lead researcher (RP) in consultation with co-authors. FGDs were concluded when saturation was reached within each subsample (boys/girls, older/younger adolescents across the two sites). Overall, 22 FGDs were conducted: 19 in schools and three at local community sites.

### Analysis

Thematic analysis was undertaken using a framework approach [20, 21]. Transcripts were coded using Nvivo 11 software. Development of the analytical framework began with a set of deductive codes derived from the research questions and background literature. The framework was refined to include codes emergent from the data. Initial codes were assigned to discrete responses comprising phrases, sentences or paragraphs communicating a relevant idea. These were ordered into categories conveying inter-related ideas. The transcripts were distributed among three authors (RP, MS, MK) for coding. RP and MS organised the data in a matrix containing codes and categories in columns, and FGDs in rows. Themes were generated by comparing and contrasting data within and across the FGDs according to age, gender and site attributes. Data triangulation was achieved initially by comparing and contrasting assignment of codes horizontally (i.e. between codes/categories) and vertically (i.e. between FGDs) within our analytic matrix. Higher-order triangulation was undertaken by scrutinizing themes across different sub-groups. Areas of agreement and disagreement have been highlighted in the narrative summary of results.

## Results

Themes have been organized into three broad categories: 1) descriptions of stress in relation to the ecological context (‘common ecological stressors’); 2) experienced reactions to stress (‘stress reactions’); and 3) commonly employed methods for coping (‘coping strategies’). A number of distinct and interrelated sub-themes have been used to elaborate differences across site, age and gender. Quotes from Hindi and Konkani have been translated into English and highlighted with an asterisk(*).

### Common ecological stressors

Table 2 presents an overview of ecological stressors across family, peer, school, community/ neighborhood domains, with key developmental challenges organized as cross-cutting themes and described under sub-themes below.

**Table 2:** Developmental challenges and interactions with contextual factors causing stress in adolescents

Developmental challenges (sub-themes)	Salient domains in adolescents’ ecological environment			
	Family	Peer	School	Community/ neighborhood
Academic pressure	High expectations; punishment for poor exam performance; insecurity regarding future career prospects.	Competition to perform well.	Excessive homework; punishment for poor exam performance; lack of guidance to improve exam performance.	Social constructions of ‘success’ that emphasise exam performance in order to progress into high-status professions.
Romantic relationships	Disapproval of romantic relationships and consequent punishment (especially for females).	Interpersonal problems stemming from relationships, including distress from break-ups and teasing from others.	Disapproval of romantic relationships.	Social derogation of romantic relationships.
Negotiating autonomy	Limits on how students are permitted to spend their time; parental control over career choices.	Challenges of connecting with others and gaining peer acceptance, while resisting deviant peer influences.	Restrictions on selection of subjects and limits on choices for vocational growth, especially in ‘non-academic’ fields such as sports and arts.	Restrictive social norms requiring adolescents to abide by family and school expectations.
Safety / victimization	Harsh/physical discipline directed at adolescents; exposure to domestic violence between parents (linked to paternal alcohol use); sexism and gender discrimination against girls, including lower access to material and financial resources and greater burden of household chores.	Bullying.	Corporeal punishment from teachers; lack of support to deal with bullying from peers.	Violence and sexual harassment (of females by males).

#### Academic pressure

Academic pressure was the most commonly identified stressor across the sample, irrespective of age, gender and site. This was largely driven by parental and teacher expectations, as well as personal ambitions. Adolescents expressed that parents were embarrassed, disappointed and would *“hate”* them due to academic underperformance. Teachers were seen as providing excessive homework, which added to the pressure. Parents and teachers often resorted to shouting, beating, and restriction of extra-curricular and recreational activities in a bid to improve adolescents’ focus on academic performance and thereby boost future career prospects. The pressure was often counterproductive, establishing a vicious cycle of guilt, low self-confidence, lack of productivity and poor performance, even driving some students to contemplate suicide.

*“Suppose [a student] studies well, and because of depression and tension he also loses his marks, and then parents shout on him why did you get less marks, then all the tension comes and the child is now in more tension, and then sometimes he makes suicide.” (Boy, 12–15 years, Goa)*

#### Romantic relationships

Adolescents frequently described emotional distress caused by challenges in forming, maintaining and ending romantic relationships, such as romantic rejection, one-sided attractions, arguments with partners, lack of money to buy gifts, break-ups and infidelity. These stressors seemed to be more pronounced in Delhi and were compounded by poor social acceptability for pre-marital relationships, especially for girls. Many girls considered romantic relationships *“bad”,* and reflected that it caused “loss of personal reputation”, “shame and embarrassment to parents”* and suggested *“poor upbringing”*. Girls also anticipated coercive responses from parents such as shouting, grounding and initiation of early marriage.

*“Where boys and girls go around together like boyfriend and girlfriend… this is not right. This will affect your parents.” (Girl, 13–16 years, Delhi)*

#### Negotiating autonomy

Older adolescents described stress stemming from limited personal freedoms, such that parents seemed to prescribe their life choices and decisions in areas such as education, employment and partners, especially in Goa.

*“In my opinion, some parents come in the group of peer pressure because they tell the students to go to a particular school, so after they get the job they would get more money.” (Boy, 13–17 years, Goa)*

Prevalent sexism and parental expectations to follow gender roles led girls, particularly in Delhi, to feel even more restricted, compounded by the additional burden of household chores. Younger adolescents were more accepting of parental influences, yet felt anxious about peer acceptance and described conflicts with friends as being particularly stressful. Older boys additionally discussed peer pressure for smoking, chewing ‘gutka’ (an inexpensive mixture of tobacco, areca nut and slaked lime), drinking alcohol and using other substances. Self-assertion was identified as key to dealing with peer pressure.

“They (peers) provoke him, taunt him that he is not capable enough to do it (take drugs), and then, if he is not mentally strong, he goes for it, and although he regrets it, he keeps doing it.” (Boy, 15-17 years, Delhi)*

#### Safety

Adolescents across both sites faced actual and threatened violence and/or victimization in their daily lives. Girls in Delhi experienced a high risk of public sexual harassment, known colloquially as ‘eve teasing,’ including both verbal and physical encounters in their neighborhoods.

*“If let’s say that a guy (in the bus) attacks you… then they (parents) will not send us to school. And no one supports us in this problem, neither friends nor teachers.” (Girl, 13–16 years, Delhi)*

Younger boys discussed being teased and bullied by older students. Boys also experienced physical punishments at home and school more often than girls. Common reasons for physical punishment were failure to complete homework, poor exam performance and disruptive classroom behavior. Further threats to safety included witnessing domestic violence and the closely related problem of alcoholism among male family members.

“I get tensed when my dad is fighting at home. I feel like doing something to myself.” (Girl, 13-16 years, Delhi)*

Additionally, younger adolescents in Delhi highlighted poverty and consequent hopelessness as stressors.

“Poor people’s financial situation is quite bad. Parents do not have a salary that can cover rent, groceries, and everything… and because of that the child also becomes depressed. He worries what would happen… because of this he doesn’t feel interested in home or school.” (Girl, 14-16 years, Delhi)*

### Stress reactions

The English terms *“tension”* and *“stress”* were used almost universally across the sample to describe everyday experiences of emotional distress. More pronounced stress reactions were also evident from the use of terms like *“mind attack”*, *“depressed”*, *“suffering”*, *“fear”* and *“sadness”*.

“Firstly, we have to face family problems at home, and we feel bad, and then we can’t even concentrate on studies (in school) … It is like a mind attack.” (Boy, 17 years, Delhi)*

*“You cannot express to another person. Means you cannot feel well and you cannot tell anyone and then you feel depressed. You feel suffocated and also cry.” (Boy, 13–15 years, Goa)*

Sudden and explosive anger, associated with shouting, throwing and breaking things, was also commonly described. Some adolescents – more often boys – resorted to hurting themselves or others when angry. Stress was also associated with irritability, arguments and fights following minor provocations, as well as loneliness and social withdrawal, which were more commonly reported by girls.

“When I get angry, I hit my brother and sister.” (Boy, 14 years, Goa)*

“When angry, we hit ourselves in front of the mirror.” (Boy, 13-15 years, Delhi)*

*“Sometimes we get angry suddenly, we can’t control on ourselves. We can’t concentrate on one thing. We get confused… Some of them, they say that I don’t want life fully, say I want to die.” (Girl, 13–15 years, Goa)*

Both boys and girls also experienced physiological reactions like loss of appetite and sleep, fever, sweating, headaches and nausea, and cognitive changes such as confusion, poor concentration, forgetfulness and intrusive ruminative thoughts.

*“So I can’t sleep properly because all the tension comes in the night.” (Girl, 11–13 years, Delhi)*

“I can’t concentrate on studies. I study, but can’t remember anything… There are many thoughts that keep coming from all sides.” (Boy, 17 years, Delhi)*

### Coping strategies

Adolescents described a range of coping mechanisms, depending on the type and intensity of stressors, perceived resources and socio-cultural norms.

#### Support seeking

Across both sites, younger adolescents and girls were more likely to seek advice and instrumental support from parents and teachers, particularly for academic difficulties and ‘ragging’ (referring to junior students being harassed, humiliated or abused by senior students [25]). Friends were generally preferred for emotional support, particularly in situations where adults were considered not to be *“open minded”* about the stressor (e.g., romantic relationships, sexual harassment).

*“Depends on how big the problem is actually. Big problem like ragging or some problem with the teachers, studies, I prefer I should tell my parents about it.” (Boy, 12–16 years, Goa)*

#### Distraction

Distraction was widely used for immediate relief from negative affect and preoccupying thoughts.

“To take my mind off the stressful things, I divert my mind to something else.” (Girl, 16-17 years, Delhi)*

“When my mood is bad, I just watch TV and eat something.” (Boy, 11-15 years, Delhi)*

#### Behavioral activation

Adolescents also participated in valued activities like spending time with friends, studying and playing with younger children.

“I meet friends and have fun. That reduces my stress.” (Boy, 15-17 years, Delhi)*

#### Escape and avoidance

Many adolescents, especially boys, took active steps to avoid confrontations with parents and teachers about academic issues. This included avoiding discussion of exam results with parents, withdrawing from other family interactions, truancy when school work was incomplete, and staying away from particular teachers.

“When schools are to declare exam results, I often go to my aunt’s place to avoid my parents.” (Boy, 12-13 years, Delhi)*

#### Self-soothing

Girls were more likely than boys to describe self-soothing strategies like yoga, meditation, deep breathing and private expressions of affect (e.g., through diary entries and crying). Students also comforted themselves through eating and sleeping.

*“And to get away from that bad feeling I cry, because, when my tears come and I cry I feel light inside.” (Girl, 11–13 years, Delhi)*

#### Problem solving

Active problem solving was relatively uncommon overall and was largely confined to older adolescents. This included a handful of instances where adolescents described specific steps of problem solving.

*“If I have a problem which is very small and I am in very bad mood, I would sit in a corner for 2-3 minutes in meditation, would think over what is the problem, what solutions I have and then I would go through the solution. If something (is) very serious, then I go to the teacher and my parents.” (Boy, 15–16 years, Goa)*

#### Prayer

In desperate times, when support was not available from other sources, some adolescents turned to prayer.

*“Sometimes… in these problems, no one is there to decide on us, then we are left very lonely… Then who will listen to us? Then we starting asking God.” (Boy, 14–15 years, Goa)*

#### Substance use

A minority of boys used substances, including tobacco, cannabis and alcohol, as a means to *“forget about the stress”* and *“reduce tension.”* However, almost all groups suggested that substance use may lead to temporary relief but would ultimately cause harm.

*“Some stress they have, they will go drink or smoke, they will think that everything is ok now I’m free from this world, and no pressure is there in their mind… They say that after drinking all our problems are solved, but instead, because of drinking they are getting more pressure, they are spoiling their health.” (Boy, 11–14 years, Goa)*

#### Suicide

Suicide was considered a last resort to find relief from severe stressors like sexual assault and rape, and severe and sustained academic pressure. Some adolescents identified depression as part of a pathway from stress to suicide.

*“So first they go in depression… and then they say that no one is talking to me at all and what will I do… no one will help me… so they then do suicide.” (Girl, 11–14 years, Delhi)*

## Discussion

We have reported one of the largest ever qualitative studies on stress and coping among adolescents in India or globally. The large sample size and inclusion of two diverse urban sites enabled us to explore commonalities and differences in adolescents’ experiences of stress and coping in depth. The findings have direct implications for developing and adapting interventions that are responsive to the dynamic interplay of age-related changes in thinking, behaviour and emotional reactivity, and the wider social ecology of adolescents’ lives.

Participating adolescents were drawn from low- and middle-income communities and experienced a variety of stressors related to family, peers, school and their wider communities/ neighborhoods. Broad terms like “tension” and “stress” and specific reactions like explosive anger, irritability and rumination were frequently used to describe stress reactions. Adolescents generally favoured emotion-focused over problem-focused coping strategies; avoidance was employed more widely than active coping. Maladaptive strategies such as substance use and attempted suicide were also mentioned to manage intense emotional reactions.

Notwithstanding differences across age, gender and sites in the relative frequency and salience afforded to different types of stressors, a common thread appeared to be the broad developmental challenge of establishing an independent social identity. This struggle is characteristic of adolescence across cultures, as adolescents attempt to establish autonomy in their romantic and other peer relationships, educational/employment transitions and other life choices [26, 27]. Extensive research from the field of developmental psychopathology has shown that social challenges in adolescence operate within interacting ecological systems, which render differences in the experience of stress and coping according to an individual’s intrinsic characteristics, the immediate physical and social environment, and broader social, political and economic conditions [28]. Within this transactional framework, stress reactions may be amplified by neurobiological processes that affect adolescents’ general predisposition to emotional reactivity [1, 29].

Our study has highlighted a number of areas in which contextual factors have a particular bearing on stress and coping for adolescents in urban India. First, adolescents experienced persistent academic pressure, notably around exam performance, which was closely related to parental aspirations for adolescents to attain high-status occupations. This is corroborated by other contemporary studies from across India, indicating how rapid social changes are causing growing differences between familial expectations and adolescents’ priorities [16, 30–32]. Relatedly, the cultural proscription against pre-marital romantic relationships was reflected in the social derogation experienced by adolescents around dating and other pre-marital relations. This was especially pronounced for girls and in Delhi, with violations feared to result in severe punishments from parents. Girls also encountered restrictive gender norms that placed a high burden on their involvement in household chores, while outside the home they faced a high risk of sexual harassment. These are further indications of how contemporary trends in Indian society may be exacerbating intergenerational stresses for adolescents [33, 34]. Boys, on the other hand, appeared to be particularly vulnerable to corporeal punishments at home and in school, a practice which continues commonly in India despite legal prohibitions [35].

We observed a general preference for emotion-focused and avoidant coping across our sample. Studies from other countries have observed a similar tendency towards emotion-focused coping among adolescents, related to perceived lack of control over everyday stressors, especially in family and school domains [36, 37]. Although avoidant coping is generally associated with worse mental health outcomes [38], approaches such as behavioral disengagement and focused distraction may be adaptive when the result is to limit exposure to harmful stressors or to re-direct attention away from negative thoughts without direct suppression [39]. However, it is otherwise notable that predominant emotion-focused and avoidant coping have been linked with self-harm [40] and substance use [9, 41]; this was also borne out in the current study.

Our findings suggest a need for interventions that focus on development of a healthy repertoire of coping skills among adolescents, and which can be applied to mitigate ecological stressors and corresponding stress reactions. Risks for suicide and substance use also require assessment and appropriate interventions. The credibility of alternative coping strategies should be accounted for while developing these interventions, especially given previous research showing significant areas of mismatch between practice elements in evidence-based psychotherapies and adolescents’ habitual coping strategies [42]. Accordingly, there is significant scope for strengthening and streamlining interventions such that constituent elements are more reflective of adolescents’ own preferences and priorities [43].

For example, efforts may be needed to balance the observed dependence on emotion-focused coping with potential enhancements in problem-focused coping. When considering how to bolster adolescents’ coping repertoire, it is notable that problem solving is one of the most common elements of evidence-based psychological interventions for a range of internalizing and externalizing problems among adolescents worldwide [44, 45], suggesting the global relevance of this core practice element. Problem solving has been widely applied using self-care and other ‘low-intensity’ modalities, which is significant in terms of designing scalable psychological interventions at low-cost [46].

In addition, systemic interventions may be required to address contextual factors that are typically beyond adolescents’ individual control, such as coercive and restrictive parenting practices [47], bullying and corporeal punishments in schools, and repressive gender norms [48]. Sustaining change at an ecological level would require the committed involvement of key sectors beyond health. As such, schools have been recommended as a promising platform for delivering mental health interventions, and healthy school environments have shown to promote mental health and well-being among adolescents [49]. In India, a recently concluded study successfully used a multi-component whole-school intervention to improve aspects of school environment that are linked with important health and well-being outcomes in adolescents [48].

We note some limitations of our study. First, we did not include participants from rural areas. On the other hand, the large sample size enabled us to explore common and divergent themes across age, gender and different urban localities within India, allowing us to reflect more confidently on the relevance to the vast and growing population of urban adolescents [7]. Second, use of FGDs for data collection may have prevented in-depth exploration of sensitive issues related to sexuality, self-harm and substance use. Third, we were unable to explore variation across socio-economic groups due to relative homogeneity in SES at each site. Finally, although detailed summaries were used, audio-recording was not permitted for two FGDs; some loss of data cannot be ruled out.

## Conclusions

This large qualitative study from India has elucidated the interplay between developmental challenges and contextual factors related to home, school, peers and socio-cultural norms in shaping adolescents’ experiences of stress and coping. The findings have direct implications for preventing adolescent mental health problems, insofar as interventions should equip adolescents with age-appropriate and ecologically valid strategies for coping with key stressors and concomitant stress reactions. Efforts to design suitable interventions should balance contextually relevant considerations with broadly applicable evidence from developmental science and the global evidence base on psychotherapies, in order to ensure optimal fit for the target demographic, locality and service resources.

## Additional Files

Additional file 1: Title: COREQ checklist. Description: Reporting of the study methods as per the COREQ guidelines for reporting qualitative studies (DOCX 17 kb)

Additional file 2: Title: FGD Guide. Description: Semi-structured guide for conducting Focus Group Discussions with adolescents (DOCX 19 kb)

## Abbreviations

LMICs: Low- and middle-income countries
