# Supplementary material for: “It is like a mind attack”: stress and coping among urban school-going adolescents in India
Source: BMC Psychol. 2019 May 28;7:31. doi: 10.1186/s40359-019-0306-z (PMC6540371; doi:10.1186/s40359-019-0306-z)
Supplement: Supplementary file 1 — Title: COREQ checklist. Description: Reporting of the study methods as per the COREQ guidelines for reporting qualitative studies (DOCX 17 kb) [file 40359_2019_306_MOESM1_ESM.docx]

Consolidated criteria for reporting qualitative studies (COREQ): 32-item checklist

| **No** | **Item** | **Description** | **Location in the manuscript (Section, pg no.)** |
| --- | --- | --- | --- |
| **Domain 1: Research team and reflexivity** |  |  |  |
| Personal Characteristics |  |  |  |
| 1. | Interviewer/facilitator | RP, MS (Other interviewers involved are acknowledged in the manuscript.) | Methods, 8 |
| 2. | Credentials | RP: MPH  MS: MPH  Other interviewers (acknowledged) held graduate, or Master’s degree or PhD in psychology or public health. | Title page |
| 3. | Occupation | RP, MS were employed with the Public Health Foundation of India. RP was a PhD candidate at the time of conducting the study. | Title page |
| 4. | Gender | RP, MS: Females; other interviewers who conducted FGDs included both males and females. | Methods, 8 |
| 5. | Experience and training | Both RP and MS had acquired basic training on qualitative research in MPH program and have more than 3 years of research experience including qualitative research. RP also had 2 weeks training on qualitative research methods including analysis at Public Health Foundation of India before the study. | Title page, Methods, 8 |
| Relationship with participants |  |  |  |
| 6. | Relationship established | Yes | Methods, 8 |
| 7. | Participant knowledge of the interviewer | Participants were briefed about the purpose of the study and written information was also provided for both adolescents and parents. The necessary ethical approvals were received before commencement of study. | Methods, 8 |
| 8. | Interviewer characteristics | There are no potential sources of bias. | - |
| **Domain 2: study design** |  |  |  |
| Theoretical framework |  |  |  |
| 9. | Methodological orientation and Theory | Thematic content analysis using the “Framework approach” | Methods, 9 |
| Participant selection |  |  |  |
| 10. | Sampling | Purposive | Methods, 7 |
| 11. | Method of approach | Researchers approached participants through announcements in school classrooms and trough visits to community based organisations working with the school going adolescents of the participating schools. | Methods, 8 |
| 12. | Sample size | 191 | Methods, 7 |
| 13. | Non-participation | All potential participants identified themselves after learning about the research program agreed for participation. | Methods, 8 |
| Setting |  |  |  |
| 14. | Setting of data collection | 19 of 22 FGDs were conducted in schools, and the remainder 2 in community settings. | Methods, 8 |
| 15. | Presence of non-participants | No. |  |
| 16. | Description of sample | A sample of 191 adolescents was drawn from 9 secondary schools in Delhi and 7 secondary schools in Goa. Overall, 108 students from Delhi (35 boys, 73 girls) and 83 students from Goa (44 boys and 39 girls) participated in the study. All participants were between 11-17 years of age. | Methods, 7 and Table 1 |
| Data collection |  |  |  |
| 17. | Interview guide | Yes, the interview guide was prepared by the authors for the purpose of this study. | Methods, 8, Additional File 2 |
| 18. | Repeat interviews | No |  |
| 19. | Audio/visual recording | Yes, we audio-recorded 20 of 22 Focus Group Discussions where permission was granted by participants. | Methods, 8 |
| 20. | Field notes | Field notes were made during the Focus Group Discussions. | Methods, 9 |
| 21. | Duration | 45-60 minutes | Methods, 8 |
| 22. | Data saturation | Data saturation was discussed within the team by reviewing the field notes. | Methods, 9 |
| 23. | Transcripts returned | No | - |
| **Domain 3: analysis and findings** |  |  |  |
| Data analysis |  |  |  |
| 24. | Number of data coders | Three | Methods, 9 |
| 25. | Description of the coding tree | Deductive and inductive codes were developed. Related codes were ordered into meaningful categories to convey inter-related ideas. | Methods, 9 |
| 26. | Derivation of themes | Yes | Methods, 9 |
| 27. | Software | Nvivo, 11. | Methods, 9 |
| 28. | Participant checking | No. | - |
| Reporting |  |  |  |
| 29. | Quotations presented | Yes, quotations from participants were used to present the findings. The age and gender of participant and site are described for every quotation. | Results, 10-17 |
| 30. | Data and findings consistent | Yes | Results, 10-17 |
| 31. | Clarity of major themes | Yes | Results, 10-17 |
| 32. | Clarity of minor themes | Yes | Results, 10-17 |
