# Supplementary material for: “It is like a mind attack”: stress and coping among urban school-going adolescents in India
Source: BMC Psychol. 2019 May 28;7:31. doi: 10.1186/s40359-019-0306-z (PMC6540371; doi:10.1186/s40359-019-0306-z)
Supplement: Supplementary file 2 — Title: FGD Guide. Description: Semi-structured guide for conducting Focus Group Discussions with adolescents (DOCX 19 kb) [file 40359_2019_306_MOESM2_ESM.docx]

**Focus Group Discussion (FGD) Guide**

Q) What does the term ‘mental health’ mean to you?

*(Probe: What is the first thing that comes to mind when you hear the term “mental health”? How is mental health important to young people’s lives?)*

Q) What are the most common mental health problems faced by young people your age?

(*Probe for tension, stress, sadness, depression, anxiety or fearfulness and low confidence, concern over the way they look, exam stress, anger issues, destructive/disruptive behaviours, bullying, attention problems, learning problems, suicidal thoughts, substance abuse, drugs, etc.*

Q) What are the most common causes of such mental health problems?

*Probe for stressors like family problems, love problems, problems with peers, studies, etc.*

Q) How do mental health/stress-related problems impact you in school, at home and with friends?

Q) How do you generally deal with these problems?

- might need to explain further using an example, which can be drawn from the discussion above on problems, and their impact.

*(Probe for coping mechanisms to address emotions, problems, and thoughts.*

*Probe for diary writing, for strategies to find causes of the problems, or information seeking, looking for solutions, seeking social support – peers, parents, teachers, others, etc.*

*Probe for strategies to manage emotions and stress, use of alcohol, drugs, violent outbursts, etc.*

*Probe for referral to a medical practitioner or a counsellor, psychologist, or psychiatrist)*

(These questions were excerpted from a longer topic guide that also covered preferences and priorities for counselling services, which were outside the scope of the current study)
